# Genome‐wide association study of multiple yield traits in a diversity panel of polyploid sugarcane (*Saccharum* spp.)

**DOI:** 10.1002/tpg2.20006

**Published:** 2020-03-20

**Authors:** Xiping Yang, Ziliang Luo, James Todd, Sushma Sood, Jianping Wang

**Affiliations:** ^1^ Guangxi Key Lab for Sugarcane Biology Guangxi Univ. Nanning Guangxi 530005 China; ^2^ Agronomy Department University of Florida Gainesville FL 32610 USA; ^3^ Sugarcane Research Unit USDA‐ARS Houma LA 70360 USA; ^4^ Sugarcane Field Station USDA, ARS Canal Point FL 33438 USA

## Abstract

Sugarcane (*Saccharum* spp.) is an important economic crop, contributing up to 80% of sugar and approximately 60% of biofuel globally. To meet the increased demand for sugar and biofuel supplies, it is critical to breed sugarcane cultivars with robust performance in yield traits. Therefore, dissection of causal DNA sequence variants is of great importance, as it provides genetic resources and fundamental information for crop improvement. In this study, we analyzed nine yield traits in a sugarcane diversity panel consisting of 308 accessions primarily selected from the World Collection of Sugarcane and Related Grasses. By genotyping the diversity panel via target enrichment sequencing, we identified a large number of sequence variants. Genome‐wide association studies between the markers and traits were conducted, taking dosages and gene actions into consideration. In total, 217 nonredundant markers and 225 candidate genes were identified to be significantly associated with the yield traits, which can serve as a comprehensive genetic resource database for future gene identification, characterization, and selection for sugarcane improvement. We further investigated runs of homozygosity (ROH) in the sugarcane diversity panel. We characterized 282 ROHs and found that the occurrence of ROHs in the genome were nonrandom and probably under selection. The ROHs were associated with total weight and dry weight, and high ROHs resulted in a decrease in the two traits. This study suggests that genomic inbreeding has led to negative impacts on sugarcane yield.

**Abbreviations**
DAPC: discriminant analysis of principal components
GWAS: genome‐wide association study
indels: insertions and deletions
NGS: next‐generation sequencing
NROH: total number of ROHs
ROH: run of homozygosity
SROH: sum total length of ROHs
SNP: single nucleotide polymorphisms
TES: target enrichment sequencing
WCSRG: world collection of sugarcane and related grasses

## 1 INTRODUCTION

As an important cash crop, sugarcane contributes up to 80% of sugar and approximately 60% of biofuel globally (Dahlquist, 2013). To meet the increasing global demand for sugar and biofuel, sugarcane harvest area in the world has increased from 22.7 to 26.8 million ha in the last ten years (Food and Agricultural Organization of the United Nations, 2016). However, cropping area for sugarcane cannot be increased indefinitely because of limited farmland area and competition with food crops. In order to improve sugarcane production sustainably, breeding sugarcane cultivars with high performance for yield traits is critical. Therefore, evaluating yield traits in sugarcane germplasm and dissecting the genetic basis of causal sequence variants are of great importance to fulfill this strategy by providing tools and genetic resources.

The *Saccharum* genus consists of two wild species (*Saccharum spontaneum* L. and *Saccharum robustum* E.W.Brandes & Jeswiet ex Grassl) and four cultivated species (*Saccharum officinarum* L., *Saccharum barberi* Jaswiet, *Saccharum sinense* Roxb., and *Saccharum edule* Hassk.) (Daniels & Roach, 1987). All the *Saccharum* species are readily intercrossed, except the sterile *S*. *edule*. Modern sugarcane hybrids were primarily derived from interspecific hybridization between *S. spontaneum* (2*n* = 40–128, *x* = 8) and *S. officinarum* (2*n* = 80, *x* = 10), followed by several backcrosses with *S. officinarum*. Therefore, modern sugarcane hybrids have a chromosome number of 100 to 130 with approximately 80% of its chromosomes from *S. officinarum* and 10% from *S. spontaneum*, with 10% being recombinant (D'Hont et al., 1996), with an estimated genome size of approximately 10 Gbp (D'Hont, 2005). Because of its huge genome size and high polyploidy level (up to 12 sets of homo(eo)logs), genetic studies of sugarcane have been extremely challenging, with relatively slower progress than many other crops.

Modern sugarcane cultivars can be traced back to only a handful of *S. spontaneum* and *S. officinarum* ancestor clones (Acevedo, Tejedor, Erazzú, Cabada, & Sopena, 2017; Deren, 1995; Lima et al., 2002). This narrow genetic base hampers sugarcane cultivar improvement for yield and tolerance. Therefore, maintenance, characterization, and utilization of genetic diversity in sugarcane germplasm collections are critical for broadening the genetic background of sugarcane for crop improvement. The World Collection of Sugarcane and Related Grasses (WCSRG), which has assembled a significant amount of sugarcane genetic diversity, is an essential resource for sustained sugarcane cultivar improvement (Nayak et al., 2014; Todd et al., 2014). The collection has ∼1200 accessions from 45 countries, including *Saccharum* germplasm and closely related grass species, with the most abundant being *S. spontaneum*, *S. officinarum*, and interspecific hybrids. This collection preserves gene resources that can presumably be used to improve yield, fiber, and abiotic and biotic stress tolerance in sugarcane breeding programs. A core germplasm collection was created by selecting representative accessions from the WCSRG (Nayak et al., 2014; Todd et al., 2014), which showed a significant amount of natural phenotypic variation and are valuable resources for identifying desirable alleles contributing to yield and tolerance for sugarcane improvement.

**Core Ideas**

- 25.3 M SNPs and 2.1 M InDels were recruited for a sugarcane diversity panel.
- Putative markers/genes associated with yield traits were identified via GWAS.
- Investigated runs of homozygosity in sugarcane.

Genome‐wide association study (GWAS) has been one of the most powerful tools for discovering causal variants for complex traits in human, animal, and plant systems. Compared with traditional linkage mapping performed in biparental populations, GWAS explores unrelated individuals in natural diversity panels with many historical and evolutionary linkage disequilibrium events being included and thus is able to achieve high resolution to the gene level (Zhu, Gore, Buckler, & Yu, 2008). In addition, multiple genes and alleles involved in the same traits can be investigated and identified. With continually decreasing costs for next‐generation sequencing (NGS) technology, GWAS has been widely applied for complex trait dissection in the genomic era. However, GWAS might not be as promising in polyploid sugarcane (with up to 12 sets of chromosomes) as it is in diploids. There are several primary challenges that could hinder the use of GWAS in sugarcane. For example, (i) the relatively high sequencing depth needed to call sequence variants because of the large genome sizes (∼10 Gbp), (ii) dosages of markers are hard to determine; and (iii) most GWAS models and software are designed for diploids, in which dosages and gene actions could not be accounted for in analyses. Hence in most cases, GWAS in polyploids treats molecular markers as diploids, which might not reflect the actual genetic effects. Since the first GWAS was conducted (Haines et al., 2005), a few GWAS analyses have been performed in sugarcane (Débibakas et al., 2014; Gouy et al., 2015; Racedo et al., 2016; Wei, Jackson, McIntyre, Aitken, & Croft, 2006; Wei et al., 2010). However, all of these studies are challenged by the difficulties mentioned above. The GWASpoly in R is software tailored for polyploids, which that can model different types of polyploid gene actions in GWAS, including additive, simplex dominant, and duplex dominant actions (Rosyara, De Jong, Douches, & Endelman, 2016). This software has been tested in a simulated tetraploid population and successfully applied in potato (*Solanum tuberosum* L.), sunflower (*Helianthus annuus* L.), orchardgrass (*Dactylis glomerata* L.), wheat (*Triticum aestivum* L.), and white clover (*Trifolium repens* L.) for GWAS (Berdugo‐Cely, Valbuena, Sánchez‐Betancourt, Barrero, & Yockteng, 2017; Bock, Kantar, Caseys, Matthey‐Doret, & Rieseberg, 2018; Inostroza et al., 2018; Phan et al., 2018; Rosyara et al., 2016; Zhao et al., 2017). However, this software has not been used for GWAS in sugarcane.

A run of homozygosity (ROH) is a continuous homozygous segment of the genome in an individual caused by haplotypes being inherited from common ancestors in the past (Ceballos, Joshi, Clark, Ramsay, & Wilson, 2018). Runs of homozygosity capture the population history of a species, such as inbreeding, a change of population size, admixtures, and so on. Run of homozygosity calling relies on high‐density genome‐wide markers (Ceballos et al., 2018). With the development of NGS technologies, large numbers of single nucleotide polymorphisms (SNPs) can be generated at a relatively low cost, making it possible to perform ROH analysis to capture the genomic regions contributing to inbreeding, to assess the breeding history, and to identify the genetic components for trait selection. Runs of homozygosity are widely applied in human and animal genetics (Ceballos et al., 2018; Christofidou et al., 2015; Peripolli et al., 2018; Sud, Cooke, Swerdlow, & Houlston, 2015; Yang et al., 2015) for detecting inbreeding and loci associated with quantitative traits or diseases. Recently, significantly greater ROHs were detected in maize (*Zea mays* L.) from the Andes, suggesting a strong ancient founder event and recent inbreeding in maize from this region (Wang et al., 2017). However, there has been no report of ROHs in sugarcane genetic research.

Previously, a diversity panel of 299 accessions selected from the WCSRG on the basis of phenotypic and genotypic data, plus nine sugarcane cultivars and breeding materials (Nayak et al., 2014; Todd et al., 2014) were deeply sequenced in their gene coding regions (Yang et al., 2019) and multiple yield related traits were evaluated with three replicates (Todd, Sandhu, Hale, Glaz, & Wang, 2017). In this study, we leveraged the phenotypic data collected earlier and the large amounts of sequence variants with a confident dosage resolution in this diversity panel called from the deep sequencing data, and performed GWAS with the GWASpoly software in sugarcane. The goals of this study were to identify the markers and candidate genes associated with biomass‐related yield traits, taking marker dosages and gene actions into consideration, and to call ROHs in the panel to identify the genomic imprints of the inbreeding history of sugarcane. The results of this study not only provided the genetic basis of multiple yield traits in sugarcane but also suggest important tools and valuable genomic resources for the sugarcane community. The new methods and concepts in this study shed light on research in sugarcane and other polyploid species.

## 2 MATERIALS AND METHODS

### 2.1 Plants in the association panel

A core sugarcane diversity panel consisting of 299 accessions were selected from the WCSRG, maintained at the USDA‐ARS Subtropical Horticulture Research Station, Miami, FL, to represent the wide genetic diversity of the WCSRG (Nayak et al., 2014; Todd et al., 2014). Nine modern sugarcane hybrids frequently used as parental materials in the USA Florida sugarcane breeding program (Supplemental Table S1) were also included in this study. In total, 308 accessions were established at USDA‐ARS Sugarcane Field Station at Canal Point, FL, in 2013 in pots (37 by 37 cm) in a randomized complete block design with three replicates. Fertilizer and water were applied to maintain healthy plants in the nursery.

### 2.2 Phenotyping and data treatment

In August 2014, nine yield traits were measured within the sugarcane diversity panel on ratoon plants, with three replicates. The evaluated traits includes stalk diameter, leaf width, leaf length, stalk number, internode length, Brix, total weight, dry weight, and water content (Todd et al., 2017). In brief, the Brix, stalk number, stalk diameter, internode length, leaf width, and leaf length of the leaf at the top visible dewlap were recorded for each plant before harvesting. Immediately prior to harvest, three or more stalks (roughly equal to 1 kg dry mass) were shredded and the total weight per pot was recorded. Shredded cane was subsequently dried at 60°C and reweighed to determine the dry weight. Water content was calculated by subtracting the dry weight from total weight.

The normality of the distributions was checked for the evaluated yield traits. For the traits not following a normal distribution, Box–Cox transformation was performed with the MASS package in R (Ripley et al., 2013) to satisfy the model assumptions. Pairwise Pearson correlations among yield traits were calculated with the Hmisc package in R version 3.0.2 (Harrell, 2018). The correlation coefficients of yield traits to sugarcane dry weight and sugar content (Brix) were partitioned into direct and indirect effects according to the method of Dewey and Lu (1959); this was conducted by the lavaan and semPlot package in R (Epskamp, 2015; Rosseel, 2012). ANOVA was performed by fitting a mixed linear model with genotypes as the random effects and replicates as the fixed effects in the nlme package in R (Pinheiro, Bates, DebRoy, Sarkar, & Team, 2009). Broad‐sense heritability (*H* ^2^) was estimated via the formula:

(1) $$H^{2}=\frac{\sigma_{g}^{2}}{\sigma_{g}^{2}+\frac{\sigma_{e}^{2}}{r}},$$

where $\sigma_{g}^{2}$ is the genetic variance, $\sigma_{e}^{2}$ is the residual variance, and *r* is the number of replicates. For each trait, the best linear unbiased predictor was estimated by modeling genotypes as the random effects and replicates as the fixed effects via the lme4 package in R (Bates, Mächler, Bolker, & Walker, 2014) and the computed best linear unbiased predictions were used for GWAS.

### 2.3 Genotyping

Target enrichment sequencing (TES) of each accession was conducted previously (Song et al., 2016; Yang et al., 2019). In brief, a set of 60,000 120‐bp oligonucleotides probes were used for TES. The probes were designed according to the sorghum [Sorghum bicolor (L.) Moench.] genome (Paterson et al., 2009) and assembled sugarcane transcripts from NCBI UniGene (ftp://ftp.ncbi.nlm.nih.gov/repository/UniGene/Saccharum_officinarum, accessed 14 Jan. 2020) and The Gene Index Project database (ftp://occams.dfci.harvard.edu/pub/bio/tgi/data/Saccharum_officinarum, accessed 14 Jan. 2020). The design density of the probes was one probe per 12 kbp, with even distribution according to the whole sorghum reference genome [see the characterization of probes in Song et al. (2016) and Yang et al. (2019)]. The captured DNA libraries were prepared by following the product manual of Agilent SureSelectXT reagents (Agilent Technologies, Santa Clara, CA) and then were indexed and cleaned with Ampure beads, quantified via quantitative polymerase chain reactionwith KAPA Library Quantification Kits (KAPA Biosystems.com), and sequenced in paired‐end mode (2 × 100 bp) with an Illumina HiSeq 2000 (Illumina, San Diego, CA).

The raw SNPs were called with a uniform ploidy model (diploid) by the Unified Genotyper implemented in Genome Analysis Tool Kit version 3.30 (McKenna et al., 2010), following a previously established pipeline (Yang et al., 2019). The SNPs were then subjected to a series of filters for each individual accession: (a) a mapping quality for SNPs of >30; (b) a base quality for SNP sites of >20; (c) minimum reads were calculated for a given SNP locus with a homozygous genotype, according to the ploidy of the accessions to ensure that single‐dose SNPs were not called as homozygotes (Yang et al., 2019, 2017) (on the basis of the calculation, minimum reads of 5, 11, 17, 23, 29, and 35 were required for ploidy levels of 2, 4, 6, 8, 10, and 12 for homozygous genotype calling, for homozygous genotype calling; otherwise, missing genotypes were assigned); (iv) for a given SNP locus with a heterozygous genotype, the sequencing depth required to separate all SNP genotypes with a probability higher than 95% at a determined ploidy was 31, 47, 70, 90 and 108 for ploidy levels of 4, 6, 8, 10 and 12, respectively. For GWASpoly analysis considering the dosage effects, after stringent filtering, the genotypes of SNPs were scaled to the highest ploidy (12) according to ratio of clean reads for the respective alleles. For example, supposing there were 10 and 110 clean reads, respectively, for the alleles A and C at a SNP locus, the genotype of the SNP would be called as ACCCCCCCCCCC. Rounding was used for noninteger ratios of clean reads. Insertions and deletions (indels) were called and transformed via the same procedures as SNPs, described above.

### 2.4 Genome‐wide association study

Discriminant analysis of principal components (DAPC), implemented in the adegent package for R, was used to infer the population structure of the accessions in this study (Jombart, 2008; Jombart, Devillard, & Balloux, 2010) for a subset of SNPs with a calling rate of ≥95%, a minor allele frequency of ≥5%, and a density of 1 SNP per 10 kbp. *K*‐means clustering was used to identify groups, where the best *k* minimized the Bayesian information criterion and was used for GWAS. GWASpoly is tailored for autopolyploids via the Q + K mixed model and could model gene actions for polyploids (Rosyara et al., 2016). Six models were used for GWAS by GWASpoly, including a general model, an additive model, two simplex dominant models (1‐dom‐ref and 1‐dom‐alt), a diplo‐general model, and a diplo‐additive model. In the additive model, effect of markers was proportional to the dosage of minor alleles, whereas heterozygotes were equivalent to one of the homozygotes for the simplex model [see Rosyara et al. (2016) for details]. Diplo‐ means that the genotypes of the markers were diploidized for the model. Sequence variants for GWAS were filtered further with a call rate of ≥95% and a minor allele frequency of ≥0.05. Only samples with less than 20% missing data were included for the GWAS. We performed GWAS runs for nine yield traits in the full sugarcane diversity panel (with 300 accessions) in addition to several subsets separately, including Subpopulation 1 with 273 accessions from *S. spontaneum*, *S. officinarum*, modern *Saccharum* hybrids, and ancient Saccharum hybrids, Subpopulation 2 with 100 accessions from *S. spontaneum*; Subpopulation 3 with 173 accessions from *S. officinarum*, modern *Saccharum* hybrids, and ancient Saccharum hybrids; and Subpopulation 4 with 162 accessions at the same ploidy level (8). The subsets of the diversity panel were used for the GWAS as well to avoid any possibility of missing significant associations created by the population structure or diverse polyploidy levels in the full set. The annotation of candidate genes with GWAS hits were obtained from the sorghum genome annotation database (Paterson et al., 2009) and their domains were annotated with InterProScan version 5.0 (Jones et al., 2014).

### 2.5 Identification of runs of homozygosity

Single nucleotide polymorphisms clearly separating homozygotes and heterozygotes (after Steps 1 and 2 of filtering, as described in Section 2.3) were used for identification of ROHs. Additional quality control for SNPs included: an individual missing rate of <10%, a SNP call rate of ≥90%, and a minor allele frequency of <1%. Runs of homozygosity were identified in each individual by Plink version 1.90b3 (Purcell et al., 2007) with the default settings and fixed scanning windows. Specifically, the parameters used to define and identify a ROH were: (i) a sliding window contained 50 sequence variants with, at most, one heterozygous call and five missing calls; (ii) for a variant to be eligible in a ROH, the hit rate of all scanning windows containing the variant must be at least 0.05; (iii)the maximum gap between two consecutive homozygous variants was 1000 kbp; (iv) a ROH had a minimum length of 1000 kbp and contained at least 100 sequence variants and at least one variant per 50 kbp on average. Linear regression was used to study the associations between ROHs and phenotypes, controlling for population structures. Associations were performed separately for the total number of ROHs (NROH), the sum total length of ROHs (SROH), and the average length of ROHs.

## 3 RESULTS

### 3.1 Correlation and path coefficient analysis of yield traits

Nine yield traits, including Brix, stalk number, stalk diameter, internode length, and width and length of the leaf at the top visible dewlap had previously been recorded for the diversity panel of 308 lines including 299 germplasm accessions and nine sugarcane clones (Todd et al., 2017). All the traits in this panel were normally distributed, except for stalk number (Supplemental Figure S1), which was transformed further. According to the pairwise Pearson's correlation coefficients for the nine traits, five pairs of traits were found to be highly correlated (*r* ≥ 0.6 or *r* ≤ –0.6, *P* < 0.0001) (Table 1). Specifically, leaf width was positively correlated with stalk diameter (*r* = 0.82) but negatively correlated with stalk number (*r* = –0.62) and stalk diameter had a negative correlation with stalk number (*r* = –0.69), suggesting that there might be competition for C resources between stalk diameter and stalk number. According to these correlations, leaf width seems to be more important for determining sugarcane stalk diameter and stalk number than leaf length. As expected, sugarcane total weight, dry weight, and water content were highly correlated, with correlation coefficients of 0.78 between dry weight and total weight and 0.92 between total weight and water content (Table 1). We further partitioned the correlation into direct effects and indirect effects to understand the inter‐relationships among these yield traits. Stalk number and internode length had high direct effects on sugarcane yield (dry weight) (Table 2), consistent with the results of for the overall correlation coefficients (Table 1). Moreover, the indirect effects were relatively low for both stalk number and internode length. The results implied that selection on high stalk number and internode length would be beneficial for increasing sugarcane yield. On the other hand, leaf width had negative direct and indirect effects on sugarcane yield, whereas stalk diameter had negative indirect effects (Table 2), which should be selected against for yield improvement. The situation is more complex for sugar content (Brix) because all the yield‐related traits, such as stalk diameter, stalk number, and leaf width, had both relatively high negative and positive effects (direct or indirect) on sugar content (Table 3). The overall correlation should be considered when selecting breeding material. To breed sugarcane cultivars with a high sugar content, these results suggested that genotypes with high stalk diameters and leaf widths but low stalk numbers should be selected.

**TABLE 1:** Pairwise Pearson's correlation coefficients and broad‐sense heritability (*H* ^2^) for the nine yield traits evaluated in the sugarcane diversity panel

	*H* ^2^	Stalk number	Stalk diameter	Internode length	Leaf width	Leaf length	Brix	Dry weight	Total weight
Stalk number	0.88	–	–	–	–	–	–	–	–
Stalk diameter	0.91	**−0.69** ***, a	–	–	–	–	–	–	–
Internode length	0.68	0.24***	−0.31***	–	–	–	–	–	–
Leaf width	0.82	**−0.62** ***	**0.82** ***	−0.37***	–	–	–	–	–
Leaf length	0.67	−0.38***	0.53***	−0.13	0.39***	–	–	–	–
Brix	0.68	−0.38***	0.54***	−0.18*	0.35***	0.2**	–	–	–
Dry weight	0.42	0.31***	−0.24***	0.34***	−0.34***	−0.06	−0.05	–	–
Total weight	0.45	−0.03	0.18*	0.19**	0.00	0.19**	0.23***	**0.78** ***	–
Water content	0.51	−0.20**	0.38***	0.07	0.19*	0.29***	0.34***	0.58***	**0.92** ***

a Bold text indicates correlation coefficients *r* ≥ 0.6 or *r* ≤ −0.6 (*P* < 0.0001).

^*^Significant at the 0.01 probability level.

^**^Significant at the 0.001 probability level.

^***^Significant at the 0.0001 probability level.

**TABLE 2:** Path coefficients showing direct effects (diagonal) and indirect effects (off‐diagonal) of six yield traits on sugarcane dry weight

	Stalk number	Stalk diameter	Internode length	Leaf width	Leaf length	Brix
Stalk number	**0.30** a	−0.21	0.07	−0.19	−0.11	−0.11
Stalk diameter	−0.10	**0.14**	−0.04	0.11	0.07	0.08
Internode length	0.06	−0.08	**0.25**	−0.02	−0.03	−0.05
Leaf width	0.14	−0.18	0.08	**−0.22**	−0.09	−0.08
Leaf length	−0.03	0.04	−0.01	0.03	**0.08**	0.02
Brix	−0.03	0.04	−0.01	0.03	0.02	**0.08**

a The values in each column showed the direct effects of the traits on sugarcane dry weight in diagonal cells and bold font and the indirect effects in off‐diagonal cells and regular font.

**TABLE 3:** Path coefficients showing direct effects (diagonal) and indirect effects (off‐diagonal) of five yield traits on sugar content (Brix)

	Stalk number	Stalk diameter	Internode length	Leaf width	Leaf length
Stalk number	**−0.05** a	0.03	−0.01	0.03	0.02
Stalk diameter	−0.57	**0.83**	−0.26	0.68	0.44
Internode length	−0.02	0.02	**−0.07**	0.01	0.01
Leaf width	0.20	−0.27	0.12	**−0.33**	−0.13
Leaf length	0.06	−0.08	0.02	−0.06	**−0.15**

a The values in each column showed the direct effects of the traits on sugar content in diagonal cells and bold font and indirect effects in off‐diagonal cells and regular font.

The broad sense heritability (ranged from 0.42 (dry weight) to 0.91 (stalk diameter) for the nine yield traits (Table 1). The total weight, dry weight, and water content had relatively low broad‐sense heritability, probably because they were complex traits combining impacts from multiple yield traits with significant environmental effects. Overall, the estimated broad‐sense heritability indicated that these yield traits were largely controlled by genetic factors. Therefore, the nine yield traits were used for GWAS analysis. Though some traits are highly correlated, GWAS may identify the same markers as being associated with the correlated traits. To maximize the discovery of associations, we conducted GWAS for each of the nine traits separately without considering the correlation first. However, after the GWAS, we summarized and presented a unique set of associated markers.

### 3.2 Sequence variants captured by TES

At present, there are two published sugarcane genomes, one for the sugarcane cultivar R570 (Garsmeur et al., 2018) and one for *S. spontaneum* (Zhang et al., 2018). We evaluated these two sugarcane genomes, in addition to the sorghum genome as a reference for the sugarcane TES data analyses. The cleaned sequence reads of 15 samples (five samples each for *S. spontaneum*, *S. officinarum*, and *Saccharum* hybrids) were separately mapped to the three available genomes. The results showed that with the sorghum genome as a reference for the sugarcane, TES read alignment outperformed that for the two sugarcane genomes, with an average of 94.9% mapped reads vs 86.4 and 72.9% when the *S. spontaneum* and R570 genomes were used as references, respectively. The uniquely mapped TES reads in the sorghum reference genome made up 91.3%, much higher than that in the *S. spontaneum* (56.3%) and R570 (62.8%) genomes (Supplemental Figure S2). Because the TES in this study were mostly from conserved genic regions, all the downstream sequence analyses, including read alignment, sequence variant calling, and annotation, used the sorghum genome as the reference.

Overall, 8.0 billion clean reads were generated after sequencing the target regions of the 308 accessions, with 7.4 billion (92.1%) of the clean reads mapped to the sorghum genome and 7.3 billion (90.7%) uniquely mapped (Yang et al., 2019). The average sequence depth of the entire diversity panel was 99×, with a range of 9× to 375×. Within the uniquely mapped reads, 25.3 million raw SNPs were identified, 9.9 million of which (39.2%) were high‐quality SNPs after stringent sample‐by‐sample filtering (Table 4). The 9.9 million SNPs included 8.6 million biallelic SNPs and 1.3 million multiallelic SNPs. The majority of the biallelic SNPs (90.2%) were rare variants (a minor allele frequency <0.05) and were thus removed from further analysis. With a SNP call rate of ≥95%, 65,000 SNPs (0.7% of the 9.9 million high‐quality SNPs) were included as markers for GWAS. Similar patterns were observed for Indels with 880,000 high‐quality indels after sample‐by‐sample filtering, of which 130,200 (14.8%) were multiallelic and the majority of the biallelic indels (562,900, 75.1% of the 880,000) were rare variants (Table 4). With the same call rate as applied to SNPs, 9900 indels were included as markers for GWAS. Therefore, a total of 74,900 biallelic DNA markers, including 65,000 SNPs and 9900 indels, were used for subsequent GWAS.

**TABLE 4:** Summary of single nucleotide polymorphisms (SNPs) and insertions and deletions (indels) identified in the sugarcane diversity panel

Sequence variants	SNPs	Indels
Raw variants	25.3 million	2.1 million
Sample‐by‐sample filtering	9.9 million	880,000
Biallelic variants	8.6 million	749,700
Remove rare variants (minor allele frequency <0.05)	840,300	186,900
Remove variants with low call rates (<95%)	65,000	9,900
Total markers for genome‐wide association study	74,900	–

According to the sorghum genome, the majority of the DNA markers used for GWAS were in genic regions (67,500, 90.1%) targeting 10,458 gene models. The number of markers per chromosome ranged from 2758 (chromosome 5) to 12,836 (chromosome 1), with an average marker distance of 9116 bp. Given the large block of linkage disequilibrium in sugarcane (∼5 cM) (Raboin, Pauquet, Butterfield, D'Hont, & Glaszmann, 2008), the marker density in this study should be high enough for an effective GWAS.

### 3.3 Inferring the population structure

**FIGURE 1:**
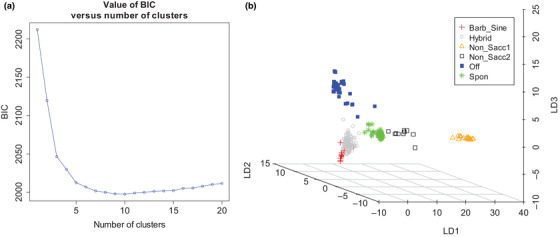
Population structure analysis of the sugarcane diversity panel based on 13,800 single nucleotide polymorphisms by discriminant analysis of the principal components. (a) Bayesian information criteria (BIC) vs. number of clusters in *k*‐means clustering. (b) Projection of the sugarcane diversity panel obtained via the first two linear discriminants (LDs). Spon, *S. spontaneum*; Off, *S. officinarum*; Hybrid, modern *Saccharum* hybrid; Barb, *S. barberi*, Sine, *S. sinense*; Non sacc, Non‐*Saccharum*

We carried out population structure analysis via DAPC on 13,800 SNPs, which were evenly distributed according to the sorghum genome (one SNP per 10 kbp) and had call rates of ≥95% in the sugarcane diversity panel. We selected *k* = 6 representing the number of population groups in the diversity panel, which was within the curve minimizing the Bayesian information criterion value (Figure 1a). Among the 308 accessions, four groups belonging to *Saccharum* were identified, with *S. spontaneum* group including 101 accessions, the *S. officinarum* group including 47 accessions, the modern *Saccharum* hybrid group including 114 accessions, and the ancient *Saccharum* hybrids group (*S. barberi* and *S. sinense*) including 17 accessions (Figure 1b and Supplemental Table S1). In addition to the *Saccharum* spp. groups, two non‐*Saccharum* groups were identified in the diversity panel as non‐*Saccharum* 1 and non‐*Saccharum* 2, with 10 and 19 accessions, respectively (Figure 1b and Supplemental Table S1). According to the WCSRG records, non‐*Saccharum* 1 most probably belonged to *Miscanthus*, whereas non‐*Saccharum* 2 belonged to *Erianthus* (Supplemental Table S1). Because of the low sequencing depth (24.3 reads per locus on average), eight accessions, including five accessions from modern *Saccharum* hybrids, two from non‐*Saccharum* 2, and one from *S. spontaneum*, which had >20% sequence variants missing, were removed from further analysis. As a result, 300 accessions from the sugarcane diversity panel were included for subsequent GWAS.

### 3.4 Genome‐wide association study

**FIGURE 2:**
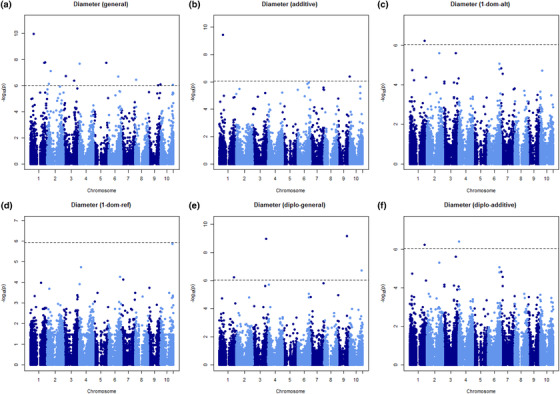
Significant marker–trait associations identified in the sugarcane diversity panel (full set) for stalk diameter (diameter) by the general model (a), the additive model (b), the 1‐dom‐alt model (c), the 1‐dom‐ref model (d), the diplo‐general model (e), and the diplo‐additive model (f)

Using a stringent threshold (*P* < 0.05 after Bonferroni correction), 217 nonredundant markers (191 SNPs and 26 indels) were identified as being significantly associated (Figure 2, Supplemental Figure S3, Supplemental Figure S4, Supplemental Figure S5, Supplemental Figure S6, Supplemental Figure S7, Supplemental Table S3). The 217 associated markers included 128, 77, 30, 13, and 58 from the full population, Subpopulations 1, Subpopulations 2, Subpopulations 3, and Subpopulations 4, respectively (see The Methods section), with some associations that overlapped (Table 5). The genome‐wide association rate was 0.55% (217 out of 74,935 = 0.29%). The number of associated markers according to the sorghum gene models was 33 in intergenic regions, 60 in introns, 48 in untranslated regions, and 66 in coding regions. For the markers in coding regions, three had high effects (stop gain or frameshift variants), 25 had moderate effects (nonsynonymous mutations), and 38 had low effects (synonymous mutations). Only 28 markers (12.9%) led to new alleles with probably different functions, whereas the rest did not change their original gene function. Among the 217 associated sequence variants, 109 were detected in at least two GWAS runs or two models, reflecting a certain level of consistence in the trait–marker association. There were nine markers associated with more than two different traits. Specifically, six markers (2P8663239, 3P66737730, 3P66744355, 4P2535140, 5ind60876916, and 10P55668582) were associated with both stalk diameter and leaf width, two markers (3P61470337 and 7P2769299) were associated with water content and total weight, and one marker was associated (3P63471814) with dry weight, water content, and total weight (Supplemental Table S3). These markers associated with multiple traits indicated that either these loci were located in or associated with pleiotropic genes or else these traits were highly correlated biochemically.

**TABLE 5:** Number of associated sequence variants identified in different runs and models of the genome‐wide association study (GWAS)

Runs^a^/Models	General	Additive	1‐dom‐alt	1‐dom‐ref	Diplo‐general	Diplo‐additive	Total
Full	99	9	25	14	35	27	128
Sacc	71	2	2	2	10	2	77
Spon	31	1	0	0	0	0	30
Hybrid	13	0	0	0	0	0	13
P8	45	6	9	7	10	7	58

a GWAS runs in the full set and several subsets. Full, all accessions in the diversity panel; Sacc, 273 accessions from *S. spontaneum*, *S. officinarum*, modern *Saccharum* hybrids, and ancient *Saccharum* hybrids; Spon, 100 accessions from *S. spontaneum*; Hybrid, 173 accessions from *S*. *officinarum*, modern *Saccharum* hybrids, and ancient *Saccharum* hybrids; P8, 162 accessions with the same ploidy level (ploidy = 8).

Among the 217 nonredundant associated sequence variants, 184 were in genic regions targeting 169 gene models and 33 were in intergenic regions according to the sorghum genome (Supplemental Table S3). To investigate the correspondence of these associated markers in genic regions between the sorghum and *Saccharum* genomes, 20 candidate trait‐associated SNPs were selected to compare their corresponding coding DNA sequences between sorghum and *S. spontaneum* via BLAST. The identity among the selected CDs between the two species was 95.3%, ranging from 91.5 to 97.5% (Supplemental Table S4) with the same annotation, supporting highly similarity in gene regions between sorghum and sugarcane, validating the transferability of the current results into sugarcane. To further investigate the genes involved in these associations, 225 nonredundant genes were obtained by retrieving the gene models hit by the associated markers and flanked by the associated markers, including 167 genes with direct GWAS hits in gene models, 56 from genomic regions flanked by GWAS hit markers, and two genes shared between the two categories (Supplemental Table S3). The average number of candidate genes was 25 for the evaluated yield traits, with a range of 2 (leaf length) to 89 (leaf width). The details of the associated markers and genes for the nine evaluated yield traits are shown in in Supplemental Table S2 and Supplemental Table S3. According to the annotations and domain analysis, among the associated genes, 10 were involved in sugar metabolism, including one (*Sobic.009G245000*) significantly associated with stalk diameter, one (*Sobic.006G278500*) with dry weight, two (*Sobic.002G279900* and *Sobic.009G063400*) with leaf width, and six (*Sobic.001G466900*, *Sobic.001G529600*, *Sobic.002G241100*, *Sobic.003G180100*, *Sobic.009G233200*, and *Sobic.009G073400*) with internode length (Supplemental Table S3). These genes encoded enzymes such as sugar‐related synthase, hydrolases, and transferase (see Supplemental Table S3 for details), which are important for regulation of C metabolism and thus contribute to biomass growth. We further searched enriched Gene Ontology categories for the 225 nonredundant genes and the genes associated for each yield trait separately. The biological process for signal transduction by phosphorylation was significantly enriched in the genes associated with stalk diameter (*P* < 0.05 adjusted for false discovery rate) (Yi, Du, & Su, 2013), indicating that signal transduction might be critical for determining stalk diameter size. We specifically focused on 27 genes associated with markers in coding regions that caused large effects (frameshift or stop gains; three genes) or moderate effects (mis‐sense or disruptive in‐frame deletions; 24 genes). The mRNA surveillance pathway was significantly enriched in the 27 associated genes (*P* < 0.05, adjusted for false discovery rate) (Yi et al., 2013), indicating that ensuring the fidelity and quality of messenger RNA has significant impacts on sugarcane yield traits.

### 3.5 Investigation of ROHs in the sugarcane diversity panel

**FIGURE 3:**
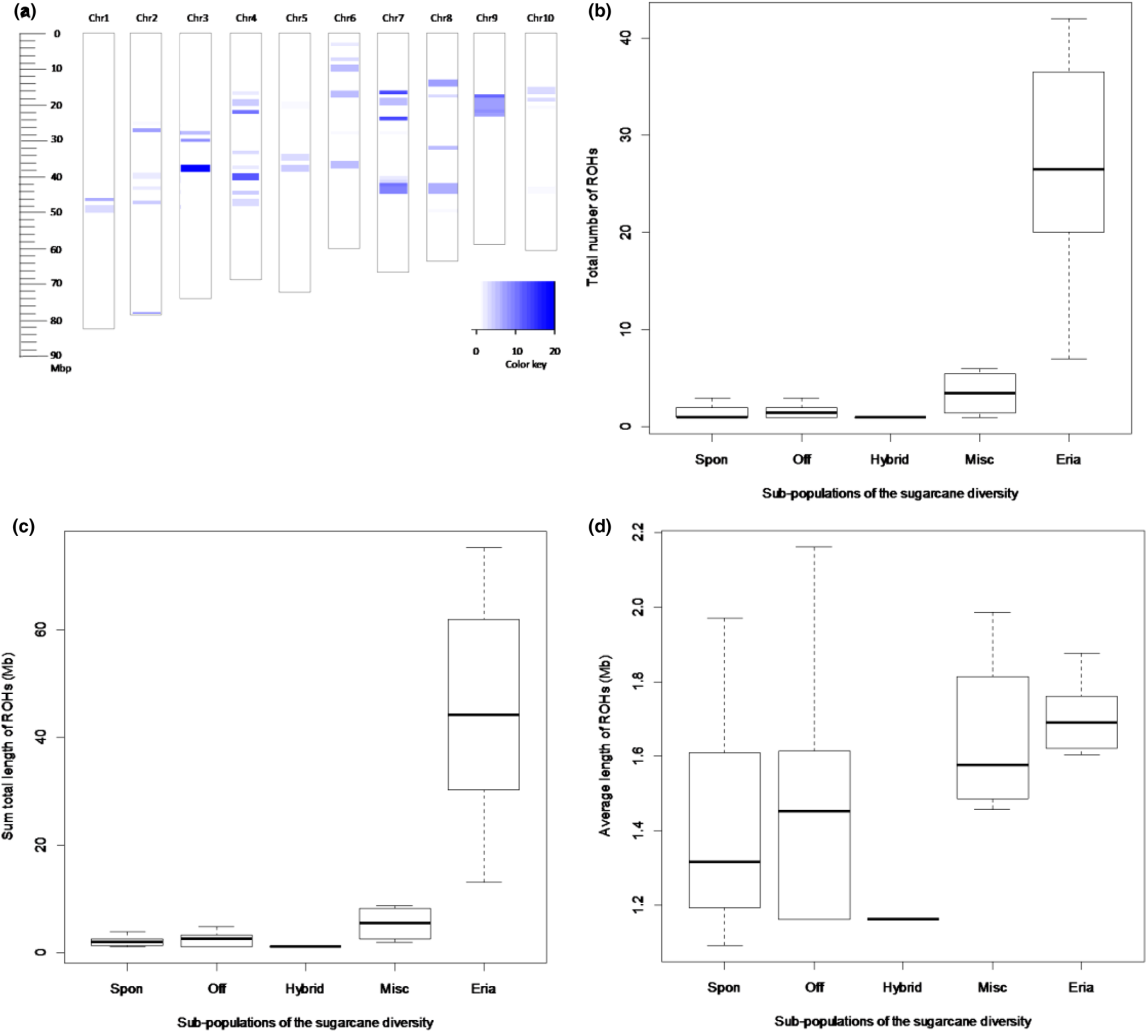
Summary of runs of homozygosity (ROHs) in the sugarcane diversity panel. (a) Distribution of common ROHs according to the sorghum genome; (b) total number of ROHs; (c) sum total length of ROHs; (d) average length of ROHs. Spon, *S. spontaneum*; Off, *S. officinarum*; Hybrid, modern *Saccharum* hybrid; Misc, *Miscanthus*; Eria, *Erianthus*

With stringent filtering, 1.07 million SNPs and 211 accessions were used for ROH identification. The total genotyping rate of the SNPs was 97.2% in the 211 accessions. In total, 282 ROHs were characterized in 46 of the 211 accessions (20.4%). The distribution of ROHs was uneven and not correlated with SNP density, with a minimum of 12 ROHs on chromosomes 1 and 5, and 57 ROHs on chromosome 7. There were 270 ROHs forming 50 pools of overlapping ROHs (the pool size ranged from 2 to 20 ROHs, with four ROHs being the most common pool size) (Figure 3a). The nonrandom distribution of ROHs indicated that the occurrence of ROHs was highly likely to have been under selection during domestication and breeding. The size of the ROHs ranged from 1.0 to 4.5 Mbp, with an average of 1.5 Mbp. The most common ROH (detected in 20 accessions) was located on chr3: 37153462 to 39398419 bp, harboring 25 genes. After Gene Ontology searches and pathway analysis (Lyne et al., 2015), the genes were enriched in five biological processes and three cellular components in photosynthesis (*P* < 0.05 adjusted for false discovery rate).

The ROH detection rate was 29.8% for the *S. spontaneum* group (out of 74 accessions), 27.8% for the *S. officinarum* group (out of 36 accessions), zero for the ancient *Saccharum* hybrids group (out of 14 accessions), 2.7% for the modern *Saccharum* hybrids group (out of 75 accessions), and 100% for both non‐*Saccharum* groups (Table 6). The average NROH and SROH was 26.9 and 45.1 Mbp for non‐*Saccharum* 2, which were significantly higher than that of any other sub‐populations in this study (*P* < 0.001) (Figure 3b, 3c and Table 6). No difference was observed for the average length of ROHs (Figure 3d and Table 6). The association analysis between the ROHs and the nine yield‐related traits showed that two yield traits, total weight and dry weight, were significantly associated with NROH and SROH (*P* < 0.05). The negative coefficients of ROHs for both traits indicated that the increase of genomic inbreeding in sugarcane had a negative influence on these two yield traits.

**TABLE 6:** Statistical summary of runs of homozygosity (ROHs) identified in the sugarcane diversity panel

	Total number of accessions	Accessions with ROHs (%)	NROH^a^ (SD)	SROH (SD)	Average ROHs (SD)
			kbp		
*S. spontaneum*	74	22 (29.8)	1.5 (0.7)	2,132.2 (908.1)	1394.3 (245.9)
*S. officinarum*	36	10 (27.8)	1.7 (0.8)	2,476.8 (1,244.8)	1460.1 (315.5)
Ancient *Saccharum* hybrids	14	0 (0)	NA	NA	NA
Modern *Saccharum* hybrids	75	2 (2.7)	1.0 (0)	1,162.1 (0.2)	1162.1 (0.2)
Non‐*Saccharum* 1	4	4 (100)	3.5 (2.4)	5,396.0 (3,267.5)	1649.7 (237.1)
Non‐*Saccharum* 2	8	8 (100)	26.9 (11.5)	45,117.7 (20,738.3)	1677.3 (144.1)
Total	211	46 (21.8)	6.1 (10.7)	9,924.5 (18,323.3)	1469.9 (269.5)

a NROH, number of runs of homozygosity; SROH, sum of total length of runs of homozygosity.

## 4 DISCUSSION

Dissection of the genetic loci controlling yield traits is of great importance for sugarcane improvement. In this study, we performed GWAS analyses in a large representative diversity panel on high‐density NGS‐based DNA markers, taking dosages and allele interactions into consideration. We identified 217 nonredundant markers and 225 genes that were significantly associated with the nine yield traits evaluated. In addition, for the first time, we investigated ROHs in sugarcane and found that ROHs were negatively associated with total weight and dry weight. This research provides valuable and novel genomic resources and tools for sugarcane breeding programs and empowers GWAS analyses in this polyploid species.

### 4.1 High‐throughput genotyping sugarcane clones empowered by TES

Sugarcane is a highly polyploid species with up to 12 sets of chromosomes and has an estimated genome size of approximately 10 Gbp (D'Hont, 2005). Because of sugarcane's nature of huge and highly polyploid genome, intensive sequencing depth is required to call genotypes accurately with allele dosage resolution, which is the prerequisite for performing GWAS with the allele dosages under consideration. Previously, we applied TES in a representative diversity panel for high‐throughput genotyping (Yang et al., 2019). By selectively sequencing mostly the gene coding regions, we achieved an average sequencing depth of 99×, which is difficult to obtain with other NGS‐enabled methods. At this sequencing depth, we could differentiate all the genotypes of the sequence variants in sugarcane, even for dodecaploid species, with up to 13 possible genotypes for biallelic sequence variants (Yang et al., 2017). After applying stringent sample‐by‐sample filtering, we obtained 74,900 markers with an average marker distance of 9 kbp according to the sorghum genome, which is much higher than that of any other published GWAS study in sugarcane. This powerful NGS‐based technology has maximized the identification of markers associated with the traits evaluated. However, we still had only 39.2% of the raw SNPs pass our stringent quality filters (mainly because of low sequence depth) and only 0.7% of the SNPs had a good call rate across the diversity panel. This significant reduction of SNP numbers during filtering informed us that the sequence depth is still the limiting factor for NGS application in this polyploid species.

### 4.2 Population structure in sugarcane

Population structure is an important factor that needs to be controlled for GWAS. Therefore, in this study, we considered appropriate software and a set of high‐quality SNPs for the structural analysis. Several software packages are available for population structure analysis for diploid species specifically, such as Structure (Pritchard, Stephens, & Donnelly, 2000) and Admixture (Alexander, Novembre, & Lange, 2009), which were not suitable for sugarcane because of its ploidy, outcrossing, and clonal propagation, which do not fit the assumptions of these software tools. We applied DAPC to capture population structures in this sugarcane diversity panel, which is a multivariate method for the analysis of the population genetic structure that does not rely on assumptions about Hardy–Weinberg equilibrium or linkage disequilibrium (Jombart et al., 2010). Compared with principal component analysis, DAPC can partition genetic variation into between‐group and within‐group components, thus achieving clear discrimination of individuals into predefined groups. With 13,800 evenly distributed high‐quality SNPs, we have inferred six groups in this diversity panel, including two non‐*Saccharum* (*Miscanthus* and *Erianthus*) and four *Saccharum* groups (*S. spontaneum*, *S. officinarum*, modern *Saccharum* hybrids, and ancient *Saccharum* hybrids), which aligned perfectly well with the biological classification of this diversity panel according to the records of the WCSRG. Moreover, DAPC could clearly separate *S*. *officinarum* from *Sacharum* hybrids, and modern *Saccharum* hybrids from ancient *Saccharum* hybrids, which are highly genetically similar, supporting the sensitivity of this method to capture the miniature population structures in sugarcane. These results were mostly consistent with the grouping of the sugarcane diversity panel of 307 accessions (Yang et al., 2019), though different SNP call settings and different subsets of SNPs were used between these two studies.

According to the quantile–quantile plots (Supplemental Figure S3, Supplemental Figure S4, Supplemental Figure S5, Supplemental Figure S6, and Supplemental Figure S7), we did not observe obvious impacts on the GWAS results resulting from population structure. To be cautious, we also ran a GWAS on four different subpopulations to avoid any possibility of missing significant associations arising from the population structure or diverse polyploidy levels in the full set. Compared with the GWAS results in the full population, 88 novel markers (out of 218 markers) were identified in the four subpopulations, reflecting their influence on declaring associations caused by population size, a certain level of population structure, or both in GWAS. This result suggested the necessity of performing GWAS in subpopulations in addition to the full population for species with a complex population structure like sugarcane.

### 4.3 Genome‐wide association study of yield traits in a sugarcane diversity panel

We selected nine yield traits and performed correlation and path coefficient analyses in a representative sugarcane diversity panel. The path coefficient analyses may suggest appropriate selection strategies for breeding programs by revealing inter‐relationship among yield traits (Dewey & Lu, 1959). For example, we recommended selecting large stalk diameter and leaf width for improving sugar content but for different reasons: a positive direct effect has been estimated for stalk diameter but an indirect effect for leaf width. The net direct effects come from the balance of contribution and competition among these components. The results of the path coefficient analysis informed us that stalk diameter might be critical for accumulating high sugar content by improving sugar storage and supporting function. Competition for C resources for leaf width and sugar content (reflected by a negative direct effect), though leaf width had a positive indirect effect on sugar content through stalk diameter. Our results also demonstrated that the same yield trait can be favored or selected against, depending on the purpose of the breeding programs. For example, genotypes with high stalk number had a high biomass yield but relatively low sugar content. Therefore, comprehensively evaluating and analyzing inter‐relationships among yield traits in a representative sugarcane diversity panel provided key information for sugarcane breeding programs.

The present study had three major improvements over previous GWAS performed in sugarcane (Débibakas et al., 2014; Gouy et al., 2015; Racedo et al., 2016; Wei et al., 2010, 2006). First, a representative diversity panel with a large number of accessions derived from WCSRG was used for this sugarcane GWAS. This diversity panel consisted of 308 accessions including 279 *Saccharum* accessions and 29 non‐*Saccharum* accessions (*Miscanthus* and *Erianthus*). The panel was not limited to sugarcane elite germplasm, as in previous studies, primarily included parental clones and sugarcane hybrids in breeding programs, and had a decent number of accessions from *Saccharum* and non‐*Saccharum* species. Therefore, the statistical power of GWAS was improved in this diversity panel. Moreover, our study has the capability to identify novel genetic resources, which might be not present in modern sugarcane cultivars because of their narrow genetic basis. More importantly, these robust novel traits could be introgressed quickly into sugarcane breeding programs through controlled crosses, especially with the help of the associated DNA markers. Second, we applied TES, an NGS‐enabled genome complexity reduction sequencing technology, to genotype this diversity panel, which proved to be very efficient for investigating sequence variants in sugarcane (Song et al., 2016). With stringent sample‐by‐sample and population‐level filtering, 74,900 markers were retained for GWAS, which was a much larger number of markers in than any of the other GWAS of sugarcane. In addition, TES achieved an average sequencing depth of 99× in this study, which allowed us to differentiate all dosages, even for dodecaploid species with up to 13 possible genotypes (Yang et al., 2017). These accurate genotypes were the prerequisite for performing GWAS with the allele dosages under consideration in this polyploid species, which was usually simplified as diploid in previous studies. Third, GWASpoly (Rosyara et al., 2016) allowed us to undertake GWAS with six different models for different gene actions. Depending on the traits and loci analyzed, different gene actions may exist. Without further experimental support, we could not evaluate which models were more suitable. To be inclusive, all six models were tested to maximize the discovery of associated sequence variants in this study.

To study a complex system like sugarcane, it is worth considering allele dosages. To minimize the possible biased effects of different polyploid levels on the GWAS analysis results, for each SNP marker locus, we used the ratio of clean reads for the respective alleles to estimate the SNP genotype with a clear dosage to standardize the results to a ploidy level of 12 for GWAS analysis. This estimation or data transformation may affect GWAS analyses by bringing in false positive associations. However, the transformation was probably the best option (at least better than simplifying the data to a diploid level) that we have for GWAS in this diversity panel via GWASpoly at present.

We identified 217 nonredundant markers and 225 genes associated with the nine yield traits. We compared our results with GWAS and linkage mappings in sugarcane. Unfortunately, such comparisons were relatively limited, since only reports on the same traits and with the associated markers or genes aligned to the sorghum genome can be compared; only a few such studies have been published. For the sugar content (Brix), two (*2P5849859* and *2P65250224*) out of three associated markers were found at a distance of 333.1 and 1631.3 kbp, respectively, from the associated gene (*Sb02g004780* and *Sb02g028450*) identified previously (Racedo et al., 2016). Recently, quantitative trait locus mapping has been conducted in a biparental population of 173 progeny with multiple quantitative trait loci detected for Brix, water content and stalk diameter (Islam et al., 2018). We had associated markers with distances of 1615.6, 6556.7, 3165.5, and 591.1 kbp from their closest markers (3SNP463 for stalk diameter, 2SNP3040 for Brix, and 1SNPUN315 and 3SNP3118 for water content), respectively. However, there were several quantitative trait loci from this mapping that were not detected in our study. Multiple reasons could have led to this discrepancy, including plant materials (a biparental population vs. a diversity panel), statistical methods (linkage mapping vs. GWAS), and the threshold for significance (logarithm of odds <3 vs. *P* < 0.05 after Bonferroni correction). We also compared our results with selection genes identified in another study (Yang et al., 2019). Eight of our associated genes are also under selection in sugarcane breeding programs. Interestingly, the candidate gene (*Sobic.009G000100*), which is also under selection and encodes SIN3 histone deacetylase protein, contained four sequence variants (one indel in intron and three SNPs in the coding region that caused one synonymous mutation and two nonsynonymous mutations), which have been identified for dry weight at least in two GWAS runs or models with –log (*P*) values ranging from 5.71 to 8.15. The combination of different haplotypes and dosages of this gene might explain the segregation of dry weight in the diversity panel. The comparisons corroborated the reliability of the GWAS results.

In this study we only focused on nine yield traits of a ratoon crop, which is the most widely used cane crop for commercial production. The yield traits were only measured in 1 yr and in a nonfield environment because the wild species *S. spontaneum* has yet to be grown in a controlled experiment. False positives were probably introduced by the environmental variance and the differences between pot and field conditions. Further validation of the results in this study through multiple years of phenotyping of the diversity panel and/or another population would be critical before the application of the identified markers or candidate genes; however, these have not been performed because of the large amount of labor, time, space and cost to be invested for such experiments. However, the current study would provide novel resources for further studies on yield traits in sugarcane and related species.

### 4.4 Runs of homozygosity in sugarcane

For the first time, we assessed ROHs in this outcrossed sugarcane by using high‐density markers. Some concerns might be raised about the accuracy of ROH detection in sugarcane. For example, loss of chromosomes or chromosome arms might make it is easy to detect ROH patterns compared with other complete chromosomes. Aneuploidy is a common event in modern *Saccharum* hybrids. However, only two ROHs were detected in modern *Saccharum* hybrids, which formed a common ROH, suggesting that loss of chromosomes or chromosome arms did not affect ROH detection in sugarcane. Another concern was the possible poor probe hybridization during TES experiments in certain genomic regions. We used Plink fixed‐window scanning methods, which require a region containing at least 100 SNPs and at least one SNP per 50 kbp to be considered as a candidate ROH; thus regions with few SNPs caused by a lack of TES reads will not pass the filtering. Therefore, we believe that the criteria for ROH detection in the current study were reasonable and the results should be indicative.

The ROH results revealed several interesting phenomena in this polyploid species. First, the investigation of ROHs could reflect the population migration, mating history and structure of sugarcane. Non‐*Saccharum* 2 had high NROH and SROH but the same average length of ROHs as other subpopulations. The SROH was correlated with NROH in non‐*Saccharum* 2 (*r* =0.99), suggesting an increase in the number of ROHs, leading to a large SROH in this species. A sudden reduction in population size might have happened in non‐*Saccharum* 2. Crosses in a population with limited number of individuals can result in a drastic increase in NROH. In addition, ROHs were not detected in ancient *Saccharum* hybrids and only two ROHs in modern *Saccharum* hybrids. Ancient *Saccharum* hybrids and modern *Saccharum* hybrids were derived by hybridization between the wild species *S. spontaneum* and the cultivated *S. officinarum* (D'Hont, Paulet, & Glaszmann, 2002). The ROH results showed that compared with their ancestors, the interspecific crosses broadened the genetic diversity and then reduced ROHs in sugarcane hybrids. The two ROHs in modern *Saccharum* hybrids (one for each accession) were located at chr07:15.9 Mbp according to the sorghum genome; these were the common ROHs detected in 12 accessions in this diversity panel. The results supported the increase of genomic inbreeding in modern sugarcane cultivars, which was probably caused by multiple backcrosses to *S. officinarum* and the sue of sugarcane parental clones with similar genetic backgrounds in breeding programs. The ROH in modern *Saccharum* hybrids was observed in chromosome 7; ROHs were popular in this chromosome (57 ROHs or 20.2% of all ROHs detected in sugarcane genomes), making ROHs relatively easy to form in modern *Saccharum* hybrids, especially in crosses made among accessions with related pedigrees. Second, the occurrence of ROHs were probably under selection because the majority of ROHs were common (96.4% were detected in at least two accessions). More interestingly, the genes landing in ROHs were enriched for certain categories with specific gene functions. In our case, the enriched genes in the most common ROHs were involved in plant photosynthesis, suggesting that the increased genomic inbreeding of the regions harboring genes related to photosynthesis would have a negative impact on sugarcane yield. Third, we observed that ROHs were significantly associated with total weight and dry weight. An increase in ROHs had a negative influence on these traits. The results further emphasize the importance of broadening the genetic basis in order to maintain sugarcane production, specifically for biomass.

### 4.5 Data availability

The cleaned target enrichment sequencing reads generated in this study were deposited into the NCBI Short Reads Archive with the accession number SRP132365. The phenotype, genotype, and population structure data used in the GWAS were deposited into Gatorcloud (https://bit.ly/2OcwsN4). The rest of the intermediate analysis data and the plant materials are available upon request.

## AUTHOR CONTRIBUTION STATEMENT

JW conceived the experiment and secured the funding for the research. XY performed the experiments and analyzed the data. JT and SS provided the yield traits. ZL helped prepare markers for GWAS. XY prepared the manuscript draft. All authors reviewed and approved this submission.

## CONFLICT OF INTEREST

The authors declare that they have no conflict of interest.

## Supporting information


**Supplemental Table S1** Details of the genotypes included in the sugarcane diversity panel.
**Supplemental Table S2** Associated DNA markers identified by GWAS in sugarcane diversity panel.
**Supplemental Table S3** Non‐redundant associated markers and candidate genes with GWAS hits.
**Supplemental Table S4** Coding DNA sequence comparisons of 20 genes harboring associated markers between sorghum and *S. spontaneum*.

**Supplemental Figure S1** Residual plots for the nine yield traits in the sugarcane diversity panel: Stalk number (Stalk), internode length (Internode), stalk diameter (Diameter), leaf width (Width), leaf length (length), Brix, total weight (Weight), dry weight (Dry), and water content (Water).

**Supplemental Figure S2** Statistics of sugarcane target enrichment sequencing clean reads mapped to three reference genomes: *S. spontaneum* (Spon), R570, and sorghum.

**Supplemental Figure S3** Quantile–quantile plots and Manhattan plots for yield traits in the sugarcane diversity panel (full set) with the general model, the additive model, the 1‐dom‐alt model, the 1‐dom‐ref model, the diplo‐general model, and the diplo‐additive model.

**Supplemental Figure S4** Quantile–quantile plots and Manhattan plots for yield traits in the sugarcane diversity panel (Subpopulation 1; Sacc set) with the general model, the additive model, the 1‐dom‐alt model, the 1‐dom‐ref model, the diplo‐general model, and the diplo‐additive model.

**Supplemental Figure S5** Quantile–quantile plots and Manhattan plots for yield traits in the sugarcane diversity panel (Subpopulation 2; Spon set) with the general model, the additive model, the 1‐dom‐alt model, the 1‐dom‐ref model, the diplo‐general model, and the diplo‐additive model.

**Supplemental Figure S6** Quantile–quantile plots and Manhattan plots for yield traits in the sugarcane diversity panel (Subpopulation 3; Hybrid set) with the general model, the additive model, the 1‐dom‐alt model, the 1‐dom‐ref model, the diplo‐general model, and the diplo‐additive model.

**Supplemental Figure S7** Quantile–quantile plots and Manhattan plots for yield traits in the sugarcane diversity panel (Subpopulation 4; P8 set) with the general model, the additive model, the 1‐dom‐alt model, the 1‐dom‐ref model, the diplo‐general model, and the diplo‐additive model.
