# Supplementary material for: Genome‐wide association study of multiple yield traits in a diversity panel of polyploid sugarcane (Saccharum spp.)
Source: Plant Genome. 2020 Mar 20;13(1):e20006. doi: 10.1002/tpg2.20006 (PMC12807312; doi:10.1002/tpg2.20006)
Supplement: Supplementary file 1 [file TPG2-13-e20006-s001.docx]

**Figure S3** Q-Q plots and Manhattan plots for yield traits in the sugarcane diversity panel (full set) using general model, additive model, 1-dom-alt model, 1-dom-ref model, diplo-general model, and diplo-additive model. Stalk = Stalk number; Diameter = Stalk diameter; Width = Leaf width; Length = Leaf length; Weight = Total weight; Dry = Dry weight; Water = Water content; Internode = Internode length.
