# Supplementary material for: Genome‐wide association study of multiple yield traits in a diversity panel of polyploid sugarcane (Saccharum spp.)
Source: Plant Genome. 2020 Mar 20;13(1):e20006. doi: 10.1002/tpg2.20006 (PMC12807312; doi:10.1002/tpg2.20006)
Supplement: Supplementary file 4 [file TPG2-13-e20006-s004.docx]

**Figure S1** Residual plots for the nine yield traits in the sugarcane diversity panel, Stalk number (Stalk); Internode length (Internode); Stalk diameter (Diameter); Leaf width (Width); Leaf length (length); Brix; Total weight (Weight); Dry weight (Dry); Water content (Water).
