# Supplementary material for: Genome‐wide association study of multiple yield traits in a diversity panel of polyploid sugarcane (Saccharum spp.)
Source: Plant Genome. 2020 Mar 20;13(1):e20006. doi: 10.1002/tpg2.20006 (PMC12807312; doi:10.1002/tpg2.20006)
Supplement: Supplementary file 6 [file TPG2-13-e20006-s007.docx]

Figure S2 Statistics of sugarcane target enrichment sequencing clean reads mapped to three reference genomes, *S. spontaneum* (Spon), R570 and sorghum. A) total mapped reads; B) uniquely mapped reads.
